# An evaluation of fertility- and migration-based policy responses to Japan’s ageing population

**DOI:** 10.1371/journal.pone.0209285

**Published:** 2018-12-19

**Authors:** Alexander J. Q. Parsons, Stuart Gilmour

**Affiliations:** 1 Department of Global Health Policy, Graduate School of Medicine, The University of Tokyo, Bunkyo, Tokyo, Japan; 2 Division of Biostatistics and Bioinformatics, Graduate School of Public Health, St. Luke’s International University, Chuo, Tokyo, Japan; Universidad del Desarrollo, CHILE

## Abstract

Japan’s ongoing struggle with rapid ageing is well known. Fertility and migration policies have both been proposed as solutions to Japan’s ageing population. We used stock flow population models to estimate the impact of hypothetical fertility and migration policy interventions on measures of aging in Japan from 2015 to 2050. We evaluated policy models based on the Old Age Dependency Ratio (OADR) they produced at the specified end date. Start dates ranged from 2020 to 2030 to assess the time horizons of individual policies. Fertility policies were found to be highly time dependent and only slowed the rate of increase of OADR. It would require a Total Fertility Rate far above replacement levels to compensate for Japan’s already aged demography. Migration policy was less time dependent. However, such measures would require unprecedented, and ultimately unrealistic, volumes of migration over coming decades in order to reduce Japan’s OADR. Our results suggest that fertility and migration based policy responses will be unable to significantly reduce Japan’s OADR or reverse Japan’s ageing population within the next few decades. Japan should focus on activating its human capital through the prolongation of working lives, increasing participation, and improving productivity within the Japanese labour force to mitigate and adapt to the inevitable effects of ageing populations.

## Introduction

Population ageing is a global phenomenon with profound implications for social stability and economic growth [1, 2]. The increased burden upon public services [3], finances [4], and wider social and familial support networks [5] in countries with aged demographics are well reported. This is also the case in Japan, where decades of low fertility and increased life expectancy have led to a significantly aged and rapidly ageing population. It is estimated that one in three people will be aged over 65 years old by 2030 [6]. This has in turn led to dramatically increased fiscal burdens, with the ratio of social security expenditure to nominal GDP being estimated to rise from 23% in 2010 to 42.9% in 2060 [7]. This trend is likely to continue for the foreseeable future.

Japan, as a forerunner of global ageing, has for several decades been attempting to respond to lower fertility, population ageing and the growing old age burden. Fertility and migration policy responses have been particularly prominent given their present low levels [6]. Efforts to ease the burden of childcare, and thus promote fertility, have included pledges to increase national childcare capacity by 800,000 places by March 2018 and gradual expansion of parental leave rights since their introduction in 1992 [8]. Migration policy is another key element of responses to ageing, with domestic companies hiring more foreign born students studying at either international or domestic universities [9] and the government recently expanded the fast-track residency scheme for highly skilled workers, reducing the minimum time period for eligibility from five years to one year [10, 11].

Despite these efforts, Japan’s population continues to age, and is one of the first countries to identify ageing as both a rural and urban issue [12]. This highlights the difficulties Japan has encountered in enacting and implementing a coherent, effective ageing strategy, especially due to ongoing “demographic momentum” [13].

However, given the rapid transition to an aged society, Japan needs to not only investigate and assess individual policy responses to ageing, but also question the fundamental viability of major policy approaches to combating the burden of ageing. Much debate about policy responses to ageing in many countries–and especially in Japan–focuses on measures to increase the birthrate or the political acceptability of immigration changes. However, if these policies are not able to reduce the burden of ageing even if implemented outside politically realistic limits, policy makers will need to prioritize alternative responses to population ageing.

For fertility and migration policies it is particularly important to assess future policy effects well in advance of their implementation time scale, due to the lag between their initial implementation and ultimate, often unexpected, social consequences [14, 15] and because their direct impact on population demographics is subject to population momentum [16]. Although the difficulties of implementing successful fertility and migration are widely reported [15, 17, 18], at present the quantitative limits and time dependency of their ability to mitigate Japan’s future old age dependency is not clearly understood.

This paper aims to analyse the impact of fertility and migration policy responses to ageing populations on population outcomes using the example of Japan by:

- Determining the realistic limits and efficacy of each set of policies by modelling a range of hypothetical policy targets
- Assessing the time dependence of these policies through the use of a range of policy start dates ranging from 2020 to 2030
- Calculating the total migrant population required to achieve optimal outcomes, and the implications of Japan’s future policy options for the wider international community

## Materials and methods

Stock flow population models were constructed using publicly available data, integrating different policy assumptions in order to assess the efficacy and time dependency of fertility and migration policies through their impact on Japan’s future population structure and Old Age Dependency Ratio (OADR). Following the example of other studies, fertility and migration projections were constructed using a single population stock and flow structure [19–21].

### Basic simulation and fertility, migration, and mortality variables

The standard demography cohort-component projection method was used as the foundation for multiple deterministic population simulations, centred around the three main demographic factors of populations: mortality, fertility, and migration [22, 23]. The basic time unit was one year. Taken together the individual scenario projections represent a plausible range of the abilities of such policies to reduce old age burden [24].

Age-specific fertility rates by individual age group were applied at each iteration to calculate the following year’s new births. This formed a flow into the next year’s stock. Age- and sex-specific mortality rates were then applied to reduce the size of the stock moving into the next year’s 1 to 100-year-old population. After application of mortality rates, the remaining population in each one-year age group was moved to the next age category. Mortality rates were held constant at 2014 levels throughout to produce as conservative an estimate of future demographic distribution as possible [25]. Mortality and cohort flow were modelled separately by sex to reflect the higher life expectancy and different late-age population distribution of women compared to men in Japan. Both fertility and mortality flows occurred even in the absence of a specified policy measure. Flows for immigration were dependent on the initiation of a policy target and the balance of in- and out-migration was held at zero in the absence of changes to migration policy.

To represent fertility policies, target total fertility rate (TFR) variables were reverse-calculated to age-specific fertility rates (ASFRs) using each group’s proportional contribution to new births in 2014 as a reference point. This allowed for increased TFR to be represented within any given year of the simulation. For simplicity and because late age of childbirth is already widespread in Japan [26], no changes in these proportions were assumed. Moreover, no extension of women’s fecund period was assumed.

To represent migration policies, simulations were integrated with a variable additional annual immigration number, reflecting the net increase in foreign born residents in a year. The annual number of immigrants to Japan in each given year was split evenly across all years in the 20 to 50 age group, reflecting the relative immobility of very young or older age groups [27], and assuming that immigration policy favours those of working age [28]. Since migration policy in the context of the simulation aims for permanent relocation into Japan, immigrants were assumed to remain in the country until their death and after arrival were subject to the same mortality and fertility rates as the Japanese population [29]. Evidence from countries such as Greece and Germany suggests that migrant fertility declines rapidly after migration, with subsequent generations eventually converging to native fertility [30, 31], and this assumption was maintained for this model. When 2014 base rates for mortality and fertility were used prior to a policy start date, the proportion rather than number of current foreign born migrants was held at a constant of 1.7% [32, 33]. Therefore, the additional annual immigration number represents an addition to the previously static migrant population.

Policy start dates were integrated into the simulation to enable the time dependency of policies to be assessed. Before the designated start date, individual projections were run through a corresponding number of annual iterations using 2014 mortality and fertility rates held constant. The proportion of foreign born residents was presumed constant at 1.7%. After this, the new policy parameters were inserted into the simulation and continued until 2050, representing the final projected target population.

### Data sources

National population data was sourced from the Japanese Ministry of Health, Labour and Welfare (MHLW) and the National Institute of Population and Social Security Research (IPSS) [34–37]. Base population data for Japan was gathered for the year 2014, including vital statistics and population size by one-year age groups. Supplementary data on international immigration and fertility trends were sourced from the OECD library and UN database [38, 39]. Due to inconsistencies in reporting between available data sets, data from the MHLW was chosen as a reference point for all analyses.

### Projection outcome

The outcome for all projections was the estimated Old Age Dependency Ratio (OADR) derived annually. Following methods commonly found in the literature [40–45], the OADR for individual projections was calculated as the population aged over 65 divided by the working age population aged 15 to 64 [46]. The OADR is frequently used as a basis for evaluating the burden of ageing when younger economically active cohorts are outnumbered by older inactive ones [47, 48].

### Statistical analysis

All simulations and analyses were conducted on R [43]. Initially, four specific scenarios were run from 2014 to 2050 to form representative policy time trends, including a base scenario, a fertility scenario, a migration scenario, and a combination policy scenario. In these scenarios all policy start dates were set to 2020. This year was chosen due to political cycles generally taking four to five years, representing the time necessary for the Japanese Diet to determine, pass, and implement any fertility and migration policy from 2015 to 2016 [49]. Simulation-specific assumptions and parameter decisions are introduced below:

- *Base scenario*: 2014 mortality and fertility rates throughout the period
- *Fertility scenario*: TFR increased to 1.7 births per woman (the OECD average in the 2000s (OECD, 2016a)) at policy start date
- *Immigration scenario*: *216*,*000 annual migrants from policy start date onwards*, equivalent to a ten percent increase in immigrants based on Japan’s 2014 immigrant population
- *Combination scenario*: Additive combination of fertility and migration scenario parameters

Population projections were also mapped onto population pyramids to better visualise the impact of different policies upon population structures. Population pyramids were produced separately using separated female and male population stocks and sex differentiated flows for mortality and fertility, whilst immigration was split evenly between the sexes.

We then performed a wider simulation analysis of the effect of changing policy settings and starting dates for both the fertility policy and the migration policy separately following basic demographic procedures [50]. For the fertility policy simulation, fertility rates were varied from TFR 1.0 to TFR 3.0 simultaneously with policy start dates varying from 2020 to 2030, and all individual projections ran from 2014 to 2050. Final OADR values in 2050 were plotted against TFR by policy start year to assess both the efficacy of individual fertility policy options in reducing old age burden and also the temporal feasibility of fertility policy as a whole.

For the migration policy simulation, the targeted additional annual immigration number was varied from 25,000 to 1,000,000 to represent a broad range of policies, reflecting a range from near zero immigration to a situation reflective of recent experiences of Germany with refugees in 2015 [51]. Policy start dates also ranged from 2020 to 2030, and the simulation was run over the same period, from 2014 to 2050. OADR values in 2050 were plotted against migration numbers by policy start year.

Based upon the example of past UN analyses, additional tests were conducted using data from the policies that commenced in 2020 to further investigate the feasibility of migration policy [52]. The final populations were used to estimate the percentage of the population that would be composed of migrants in 2050. This data was plotted to show the percentage of foreign born people Japan would have to accommodate in order to achieve any desired OADR within the target ranges [53].

The percentage of foreign born migrants necessary to retain the OADR at 2015 levels in 2050 was then plotted along with 2015 migration data from all OECD countries, the most recent year for which data is available [54], to illustrate the potential magnitude of any major future immigration policy intended to hold OADR static at current levels, if that policy started in 2020.

In order to assess the overall feasibility of immigration policy within the wider global context of population ageing, this amount was converted into a point estimate percentage of the current flow of migrants coming from Japan’s main suppliers of foreign born workers: Brazil, China, the Philippines, and South Korea [54]. This population constitutes 77% of all foreign born people in Japan and their combined population has constituted the majority of foreigners in Japan since at least 1990 in part due to their close cultural and economic links [55]. Whilst future migrants’ country of origin may differ, it is likely that these countries will remain major providers of migrants in years to come and would be crucial to any future migration based policy responses to ageing populations in Japan.

## Results

### Representative policy time trends

**Fig 1:**
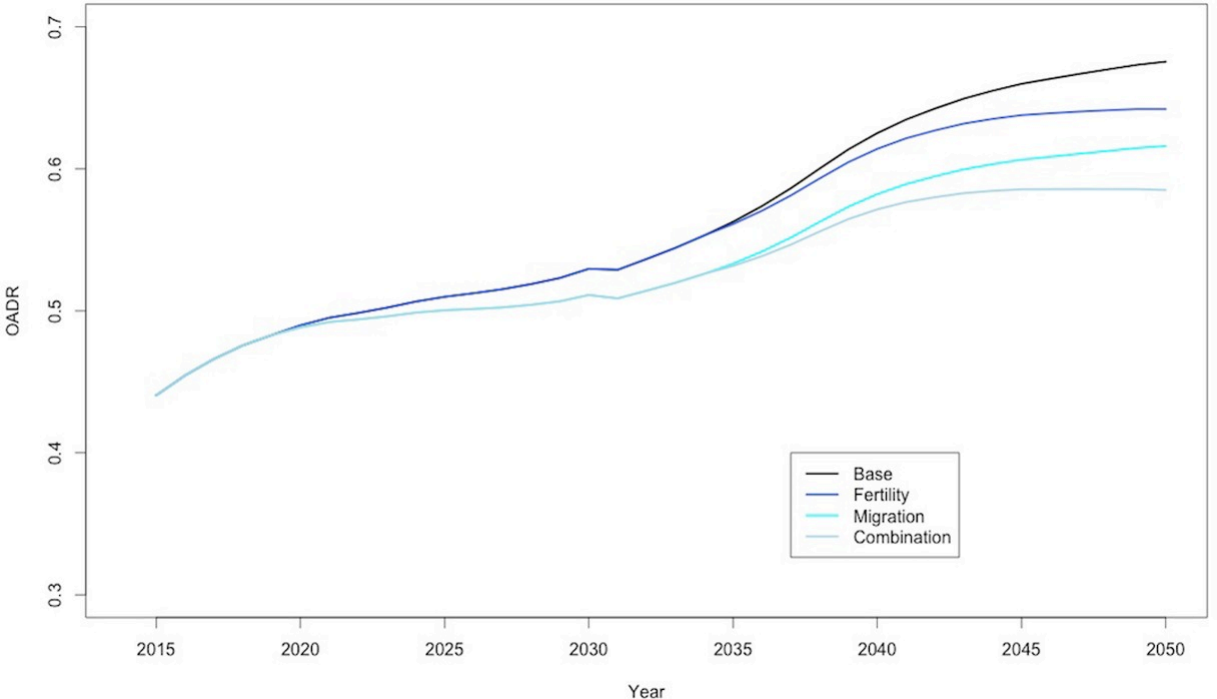
Representative policy time trends, 2015–2050. Base scenario held 2014 mortality, fertility, and migrant population percentage constant. Fertility scenario increased TFR to 1.7 from 2020. Migration scenario introduced 216,000 additional annual immigrants from 2020. Combination scenario applied variables of both fertility and migration scenarios from 2020.

The Japanese population in 2014 was 127,078,000 with an OADR of 0.42. The base scenario, where 2014 rates were held constant until 2050, produced a population of 96,665,812 and an OADR of 0.68. Fig 1 shows time trends for the four representative scenarios from 2015 onwards. A combination policy aiming for both increased fertility rates amongst women and increased annual immigration was shown to be most effective at lowering OADR over the period. This combined policy intervention was predicted to produce an OADR of 0.59 in 2050, compared to an OADR value of 0.68 produced by the base scenario.

Migration and combination policies produced an immediate reduction in the rate of increase of OADR, though were unable to reverse the overall increasing trend. Fertility based policies were slow to impact OADR due to the required time for infants to mature and enter the productive age category. Policy measures starting in 2020 only began to change the OADR in 2035.

### Efficacy and time dependency of fertility policy

**Fig 2:**
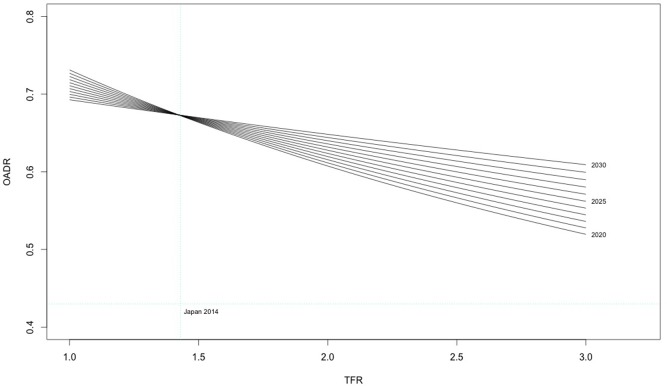
Fertility model 2050.

Fig 2 shows the fertility policy simulation, which highlights that fertility policy interventions are highly time dependent. Each line represents the OADR for a different range of TFR settings from a specified policy start date. Japan’s 2014 OADR (0.43) and TFR (1.4) are plotted on the graph in blue for reference. The earlier the policy start date the larger the possible reduction in OADR by 2050. Moreover, this relationship increases in strength with increases in TFR, as shown by the fan shape of the chart. The simulation found that a policy of unchanging fertility rates would result in an OADR of 0.68 in 2050. However, this simulation showed that even with an aggressive pro-natal policy Japan will be unable to regain its 2014 OADR level for at least a generation. Even with fertility rates above the 2.1 replacement level, OADR values will be far removed from present rates, and even a rapid return to replacement level fertility, given by a TFR of 2.1, shows only modest potential reductions in OADR at 2050: returning to replacement level TFR in 2020 would result in an OADR of 0.60, whilst a 2030 start would lead to a value of 0.64. Both values are higher than the 2014 TFR but only slightly lower than the OADR of 0.68 produced by constant rates without intervention in the base projection.

### Efficacy and time dependency of migration policy

**Fig 3:**
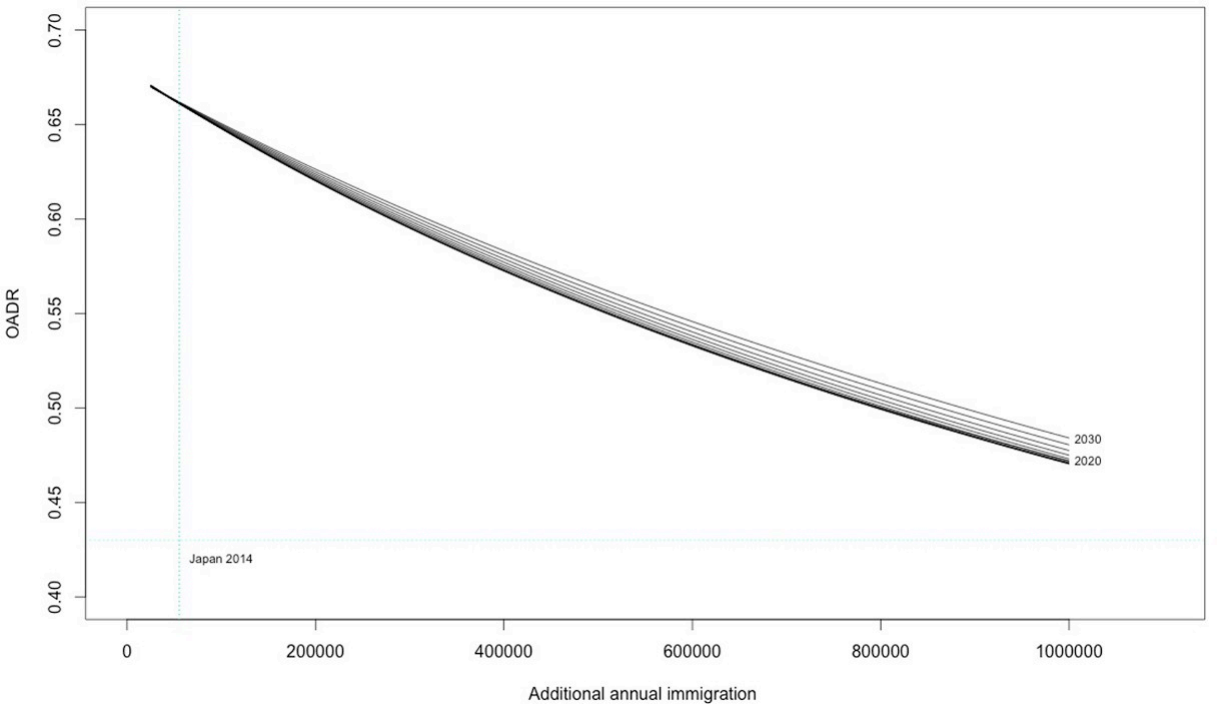
Migration model 2050.

Fig 3 displays the migration simulation. It shows that migration policy is considerably less dependent on start date and is more responsive to policy settings, with the largest difference between OADR values by starting year only 0.1 between 2020 and 2030 at the policy setting of an additional 1,000,000 migrants per year. The steeper slope of the curves shows the greater efficacy of migration policy in reducing OADR, although significant reductions can only be seen for increases of 300,000 migrants or more annually. Overall there was a greater potential reduction in OADR compared to fertility, with a low value of 0.47, which indicates that an extremely large migrant intake could hold Japan’s demographic structure at 2014 levels.

**Fig 4:**
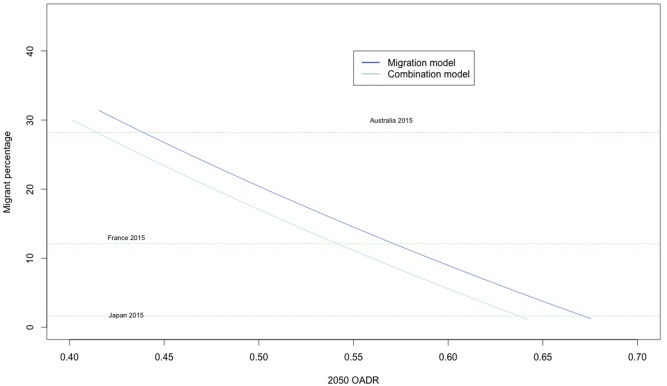
Required migrant populations by 2050 OADR. Assuming a 2020 policy start date. Prior to start date mortality and fertility were held constant at 2014 rates. Combination scenario increases TFR to 1.7 after policy start date alongside additional migrant number.

Fig 4 is based on the data from the migration and combination scenarios. It shows the proportion of foreign born migrants Japan’s population required in 2050 to achieve specific OADR targets, assuming a 2020 start date. The 2015 foreign born population percentage of several OECD countries (including Japan) are plotted as horizontal lines for reference.

Extrapolation of these figures shows that if policy measures were to begin in 2020, Japan would have to increase the proportion of its foreign born population dramatically in order to lower its OADR even slightly, whether or not fertility was to rise.

**Fig 5:**
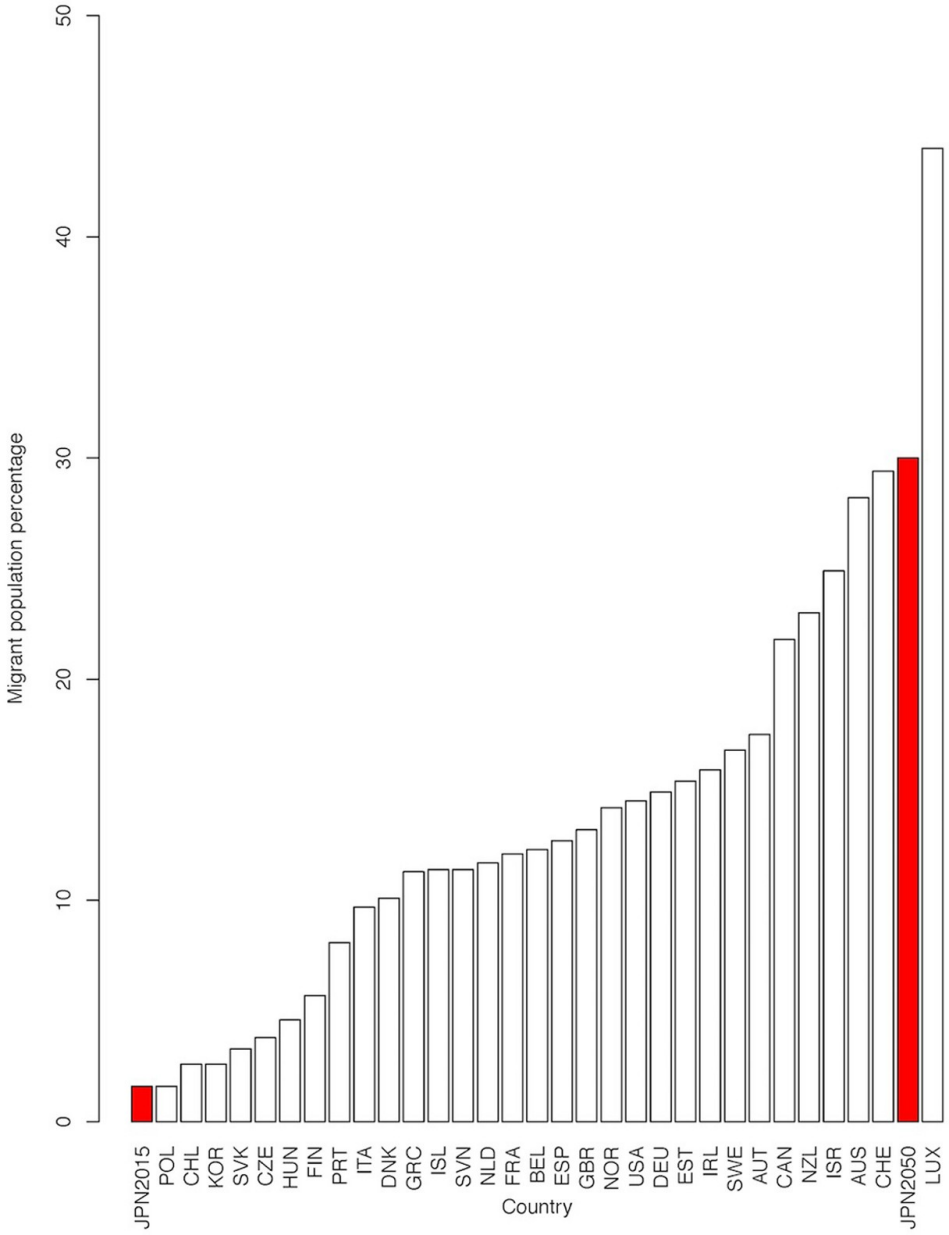
OECD migrant population comparisons if Japan maintains 2015 OADR in 2050. Comparison of OECD countries’ foreign born populations for 2015 Japan’s real 2015 value and hypothetical 2050 value are both displayed. 2050 predicted value reflects the necessary percentage of migrants to achieve the same OADR as 2015 in 2050. Data source: OECD library, 2016.

For instance, based on a purely migration-focussed policy, Japan would have to achieve a population composed of 30% foreign-born migrants to retain its 2015 OADR of 0.43. Fig 5 shows this value plotted against data from other OECD countries in 2015 [41]. According to OECD data, Japan in 2015 had the second lowest percentage of migrants in the population at 1.6%. An increase to 30% would be equivalent to Japan jumping to the second highest foreign migrant percentage in the OECD, after Luxembourg. Note that the percentages in Fig 5 would be larger and potentially have a different national ranking if children of immigrants are included in the calculation of the proportion of migrants.

The four largest origin countries for migrants to Japan are Brazil, China, the Philippines, and South Korea. By 2015 a total of 18,752,249 of their citizens were based overseas, some 1,567,369 of whom were living in Japan [40]. To achieve a migrant intake equivalent to 30% of the Japanese population in 2050 and retain the OADR at 2015 levels, a total migrant population of 44,087,269 would have to be gained between 2020 and 2050. The current supply of migrants to Japan from the main origin countries in 2015 represents only 3.56% of the total amount that would be necessary in 2050. Moreover, this amount is equivalent to 235% of the current migrant population of the four main donor countries. Given global trends in development and low fertility it seems unlikely that the future supply of migrants will keep up with such demand, especially as countries with ageing populations increasingly compete with one another to attain human capital.

## Discussion

This study assessed several policy strategies for ageing populations and showed that neither fertility nor migration policy will be able to noticeably lower old age dependency in Japan over the coming decades, even if aggressive policies are implemented within a very short time frame. Population momentum [41] combined with both domestic and international realities [15, 40] makes reversal of Japan’s demographic trends an unrealistic policy objective.

In the case of fertility, this study found that a rapid, significant increase in Japan’s TFR to levels well above the replacement rate of 2.1 would be necessary to maintain an OADR even close to current levels. To date no country has managed to produce such an increase intentionally, no matter how fervently they push a pro-natalist agenda. For example, South Korea pursued the ambitious First Basic Plan for Low Fertility and Aged Society between 2006 and 2010 but was unable to raise its national TFR significantly [56]. Whilst fertility policy suffers from considerable time lags, any increase in TFR would constitute at least some return to historical dependency ratios. The results showed that given enough time a modest baby boom could be initiated through Japan meeting the OECD average TFR.

Most importantly, aggressive pro-natal action would entail a significant burden upon Japanese women who are already often forced to choose between motherhood and participation in the labour market [57]. This potential trade-off is central to any debate on fertility policy since both outcomes are considered vital for responding to ageing populations. This issue is also exacerbated by the low rates of extra-marital childbirth and cohabitation in Japan, which are amongst the lowest in the world, limiting the socially acceptable avenues within which women may feel at ease to start a family [58]. Japan has already started to offer bonuses for childbirth, increased parental leave, and subsidized childcare over recent years which, despite a slight rise in TFR in the last decade, have had only a limited effect on the overall number of new-borns [59]. It is therefore unclear how much more can be done to try and raise TFR under the current range of available, realistic policy options.

Migration policy was shown to be less time dependent, suggesting that consistency in immigration targets is more important than actual start date. Whilst significant reductions in old age dependency are possible through a large influx of immigrants it seems unlikely that such a trend could be maintained both at a domestic socio-political level and also within an international environment of increased competition for skilled foreign workers [60]. This study also showed that projected demand for migrants would far surpass current supply in any scenario that significantly reduced future OADR. In some regards our results were similar to those seen in a recent Eurostat paper [61], which observed that only through migration at levels similar to European countries could Japan avoid rapid ageing and that this would result in a radical diversification of the population. Moreover, additional human capital does not guarantee economic dividends and may place new burdens upon public services [29, 62].

Japan is a highly homogeneous society contending with a rapidly ageing population. If the government decided to commit to the reversal of ageing populations the policy reality for Japanese citizens would be dramatic, requiring foreign-born populations of unprecedented sizes. Furthermore, special policy measures and inducements would likely be required to attract large migrant flows in competition with other countries, the cost and feasibility of which are not yet well understood. This large expansion of migration numbers would happen in the context of rapid development in countries with current net outwards migration. Such countries will begin to retain more of their human capital, resulting in the overall global supply of migrants dropping [63, 64]. In this scenario it is likely that Japan would face increasing competition for migrants both from the migrants’ own origin countries, or from countries that are better able to target migrant populations through shared language or political ties. As such the capacity for the government to act upon a bold migration policy is likely limited and not feasible as a sole policy solution, regardless of the domestic political favourability of migration policy.

There were several limitations to this analysis which may reduce the scope of our findings. Fertility models used a single start date, creating exaggerated jumps in fertility. However, this would have most impact in scenarios where the target TFR was already unrealistically high. Realistic policy targets would expect a gradual increase in TFR, though the results of more nuanced changes in TFR would broadly converge with 2050 predictions presented in this paper. Given this, we chose to use step changes in TFR because these highlight most clearly the magnitude of policy changes required to achieve any moderation of the trend in OADR in Japan. All analyses were conducted at a national population level and did not address geographical differences in both fertility and immigration. This is important in Japan where rural areas are disproportionality ageing due to depopulation via youth flight, already low local fertility, and an inability to attract foreign residents [63]. Further micro-level analyses could illuminate challenges at a regional level and prove useful to policy makers at these regional centres. A more detailed probabilistic model could have been integrated to account for possible changes in average life expectancy and advances in infertility treatment, allowing for the extension of fecundity in women, but it is likely that such a model would not significantly change the results presented here, as increases in life expectancy are likely to have a far greater effect on ageing than extensions of fecundity will have on fertility. We assumed a flat age distribution of immigrants, under the assumption that the current youthful distribution of migrants to Japan cannot be maintained over the long term as the available population of migrants in neighbouring countries is exhausted and those countries themselves age, but this is likely to only present a second-order effect on the estimate of the OADR, since OADR is independent of the specific age distribution within the 15–64 age group. We also assumed that new migrant populations would be approximately evenly distributed by sex, although the specific sex distribution of any migration policy would depend upon its goals. This assumption would likely have minimal impact on long term estimates of the OADR because it is not dependent on the specific sex distribution within the 15–64 age group.

Several Japanese public figures endorse pro-natal policy and/or liberal migration policy as the most effective policy solutions to ageing populations. Prime Minister Abe is reported to want to raise the national birth rate to 1.8 [65] and in the past several members of his cabinet have voiced support for relaxing barriers to immigration, including Shigeru Ishiba, Minister in charge of Overcoming Population Decline and Vitalizing Local Economy in Japan [62]. However, these pro-natal policies place strong expectations on women’s personal and lifestyle choices, often while ignoring the known and complex social, policy and labour market reasons for the decline of fertility rates in Japan [66]. Whilst other studies and reports have highlighted the difficulties of implementing such measures [67, 68], relatively few have concluded that pursuing such policies proactively may be futile and represent a waste of both time and resources which might otherwise be spent preparing for the inevitable outcome of population decline.

Recent research has embraced adaptation to rather than reversal of the ageing process. For instance, researchers have noted that the implications of low fertility vary due to the nature of wealth flows, with public finances benefiting from high fertility whilst standards of living and private intergenerational wealth flows flourish in low fertility environments [4]. Novel measurements of dependency which go beyond simple demographic metrics to include healthy life expectancy [10], labour participation amongst older age populations [43, 44], changes in workforce structure, women’s participation and migration dynamics [46], represent opportunities to show how far-sighted strategy revolving around technological progress and labour productivity may hold the real key to coping with the burden posed by increasingly aged populations.

## Conclusion

This study has shown that countries facing ageing populations will need to implement rapid, aggressive and likely unrealistic policies if they aim to reverse the effect of ageing through direct demographic interventions. Rather than focus on these policies, Japan and other countries experiencing ageing would do best to prioritise a policy of adaptation through raised labour force productivity, greater workforce participation, and active investment in technological innovation. This combined with a limited pro-natal, pro-migrant policy should enable populations to adapt to the challenges posed by shrinking populations. Japan is blessed with an educated and innovative population and robust infrastructure and social care systems. As such it may be the ideal country to highlight in demographics that quantity certainly does not equal quality; and that an ambitious and nuanced strategy of adaptation may serve ageing countries better than short term attempts to reverse what is ultimately an inevitable consequence of human development.

## Supporting information

S1 File The basic population data. (CSV)

S2 File Data on sources of migration to Japan. (CSV)

S3 File Total available migrant population from source nations. (CSV)

S4 File R script to run the model. (R)
